# Proteomic analysis of the *Theileria annulata* schizont

**DOI:** 10.1016/j.ijpara.2012.10.017

**Published:** 2013-02

**Authors:** M. Witschi, D. Xia, S. Sanderson, M. Baumgartner, J.M. Wastling, D.A.E. Dobbelaere

**Affiliations:** a Division of Molecular Pathobiology, DCR-VPH, Vetsuisse Faculty, University of Bern, CH-3012 Bern, Switzerland; b Department of Infection Biology, Institute of Infection and Global Health & School of Veterinary Science, University of Liverpool, Liverpool L69 7ZJ, UK

**Keywords:** Apicomplexa, Detergent, Triton X-114, Mass spectrometry, Proteome, *Theileria*, Transformation, Transmembrane domain

## Abstract

The apicomplexan parasite, *Theileria annulata*, is the causative agent of tropical theileriosis, a devastating lymphoproliferative disease of cattle. The schizont stage transforms bovine leukocytes and provides an intriguing model to study host/pathogen interactions. The genome of *T. annulata* has been sequenced and transcriptomic data are rapidly accumulating. In contrast, little is known about the proteome of the schizont, the pathogenic, transforming life cycle stage of the parasite. Using one-dimensional (1-D) gel LC-MS/MS, a proteomic analysis of purified *T. annulata* schizonts was carried out. In whole parasite lysates, 645 proteins were identified. Proteins with transmembrane domains (TMDs) were under-represented and no proteins with more than four TMDs could be detected. To tackle this problem, Triton X-114 treatment was applied, which facilitates the extraction of membrane proteins, followed by 1-D gel LC-MS/MS. This resulted in the identification of an additional 153 proteins. Half of those had one or more TMD and 30 proteins with more than four TMDs were identified. This demonstrates that Triton X-114 treatment can provide a valuable additional tool for the identification of new membrane proteins in proteomic studies. With two exceptions, all proteins involved in glycolysis and the citric acid cycle were identified. For at least 29% of identified proteins, the corresponding transcripts were not present in the existing expressed sequence tag databases. The proteomics data were integrated into the publicly accessible database resource at EuPathDB (www.eupathdb.org) so that mass spectrometry-based protein expression evidence for *T. annulata* can be queried alongside transcriptional and other genomics data available for these parasites.

## Graphical abstract

**Graphical abstract:**

**Figure:**
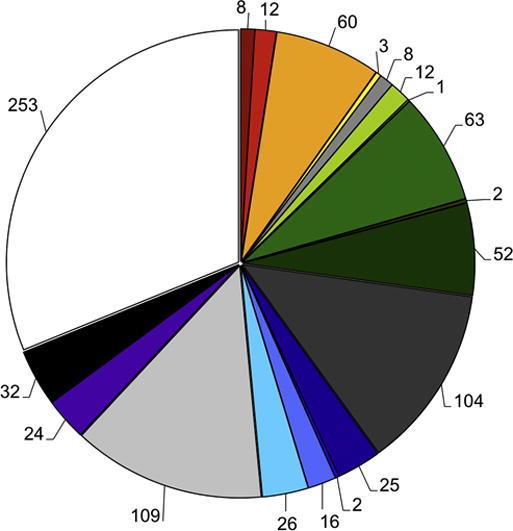

**Highlights:**

► Proteins (812) were identified in a proteomic analysis of purified *Theileria annulata* schizonts. ► Several proteins previously thought to be sporozoite-specific were also identified. ► Membrane proteins were considerably enriched by Triton X-114 extraction. ► With two exceptions, all proteins involved in glycolysis and the citric acid cycle were identified.

## 1 Introduction

*Theileria* parasites belong to the Apicomplexa, are transmitted by ticks and cause diseases with significant economic impact in a range of domestic livestock, including large and small ruminants (Bishop et al., 2009). Unique among eukaryotic organisms, several members of the genus *Theileria* possess the ability to transform the cells they infect. Transformation is achieved by interfering with pivotal host cell signalling pathways that regulate proliferation and cell survival (reviewed in Dobbelaere and Baumgartner, 2009; Chaussepied and Langsley, 2011). This is facilitated by the fact that, immediately following entry into the leukocyte, the host cell membrane surrounding the invading sporozoite is dissolved, leaving the developing schizont free in the cytoplasm where it can interact with the host cell cytoskeleton and regulatory components of signalling pathways. Analysis of leukocyte gene expression networks provided evidence that the parasite establishes tight control over pathways associated with cellular activation by modulating reception of extrinsic stimuli and by significantly altering the expression outcome of genes targeted by infection-activated transcription factors (Durrani et al., 2012). Furthermore, at each host cell mitosis and cytokinesis, the schizont co-opts the host’s astral and central spindle microtubules, ensuring its equal distribution between the two daughter cells (Hulliger et al., 1964; von Schubert et al., 2010). Although significant progress has been made in identifying the host cell pathways that are directly or indirectly targeted by the parasite, little progress has been made in pinning down the parasite proteins that are involved in host cell transformation. This is largely due to the fact that knowledge on the spectrum of *Theileria* schizont proteins that could function as participants in the transformation process is very scarce. As an important step in that direction, the genomes of two transforming *Theileria* parasites, *Theileria parva* and *Theileria annulata*, have been sequenced and annotated, providing a wealth of new information on these organisms (Gardner et al., 2005; Pain et al., 2005; Shiels et al., 2006). Analysis of the *T. parva* schizont transcriptome using Massive Parallel Signature Sequencing (MPSS) provided evidence for the transcription of 2,533 of the 4,036 predicted protein coding genes of *T. parva* (Bishop et al., 2005; Shah et al., 2006), 405 of which encoded proteins with a predicted signal peptide, suggesting they could be secreted. Of 3,794 *T. annulata* genes analysed, 628 were predicted to encode proteins with a signal peptide (B. Shiels and W. Weir, personal communication). A more recent study also reported on the evolution and diversity of *T. annulata* secretome genes (Weir et al., 2010) and Shiels et al. (2006) generated a list of *Theileria* proteins containing a predicted signal peptide and which could be involved in manipulating the host cell phenotype. As is the case for many parasites, the majority of the predicted *Theileria* proteins (75%) are classified as hypothetical proteins that show no identity to known proteins in public databases (Gardner et al., 2005; Pain et al., 2005). In the case of *T. annulata*, an expressed sequence tag (EST) library prepared from mRNA isolated from purified schizonts has been generated and 1,407 *T. annulata* genes (representing 37% of predicted genes) could be identified that showed cDNA hits when screened in silico against this library (Pain et al., 2005). As a first step towards characterising the *T. annulata* schizont proteome, a one-dimensional (1-D) gel-based analysis of schizont proteins followed by MS was carried out, using purified parasites as starting material. This first analysis underpins the notion that proteomic analysis is an indispensable tool to study the biology of *Theileria*/host cell interactions.

## 2 Materials and methods

### 2.1 Parasite strain, culture conditions and parasite purification

*Theileria annulata* – infected macrophages (TaC12, strain Ankara; Shiels et al., 1992) were cultured in Leibovitz 15 medium (Gibco, Switzerland) supplemented with 10% FCS (Bioconcept, Switzerland), 10 mM HEPES, pH 7.2 (Merck, Switzerland), 2 mM l-glutamine (Gibco), 70 μM β-mercaptoethanol (Merck, Switzerland), 100 μ/ml of penicillin and 100 μg/ml of streptomycin (Lonza, Switzerland). Schizonts were purified as previously described (Baumgartner et al., 1999). Briefly, TaC12 cells were incubated for 2 h with nocodazole to depolymerize microtubules. Cells were then treated with trypsin-activated aerolysin on ice. After removing excess aerolysin, cells were exposed to a temperature of 37 °C to stimulate toxin-mediated permeabilization of the host cell plasma membrane. Permeabilization was monitored using Trypan blue exclusion. Schizonts were separated from host cell debris using Percoll gradient centrifugation.

### 2.2 Generation of whole parasite lysates (WPL), protein digestion and MS

Purified parasites were solubilised in 40 μl of Lämmli buffer with four cycles of boiling at 95 °C and vortexing for 5 min. Samples were centrifuged at 12,000*g* for 5 min. The supernatant was run on a 16 cm 12% v/v SDS–polyacrylamide gel at 16 mA for the stacking gel (for ∼1 h) and at 24 mA for the separating gel (for ∼6 h). Gels were fixed in 40% v/v ethanol, 10% acetic acid overnight at room temperature (RT), rinsed in distilled deionised water and stained with colloidal Coomassie (20% methanol, 0.1% W/v Coomassie Brilliant Blue G250, 1% v/v H_3_PO_4_ (85%), 10% w/v (NH_4_)_2_SO_4_ and destained in distilled deionised water. Lanes were cut, using a scalpel, into continuous slices of approximately 1 mm thickness and stored in 1% acetic acid at 20 °C. Gel plugs were fully destained in 50 mM NH_4_HCO_3_, 50% v/v acetonitrile at 37 °C, and incubated at 37 °C with 10 mM DTT, 100 mM NH_4_HCO_3_ for 30 min and subsequently with 100 mM iodoacetamide, 100 mM NH_4_HCO_3_ for 1 h in the dark. Gel pieces were dehydrated with acetonitrile and proteolytic in-gel digestion performed at 37 °C by adding 10 μl of 10 ng/ml of sequencing grade trypsin (in 25 mM NH_4_HCO_3_). After 45 min, 25 mM NH_4_HCO_3_ was added to cover the gel pieces and the incubation was carried out overnight at 37 °C. Samples were stored at −20 °C until used for LC-MS/MS.

As the aim of this study was to discover and validate *T. annulata* schizont proteins, rather than comparing proteins or studying differential expression, biological replicates were not produced. On this particular MS platform, running replicates to increase the number of identifications typically results in improvements of no more than 10% and often considerably less. Instead, we focussed on improving the isolation and identification of membrane proteins (see Sections 2.3 and 2.4).

### 2.3 Triton X-114 extraction of schizont proteins

Purified *T. annulata* schizonts were lysed and homogenised for 1 h at 4 °C in 1 ml of lysis buffer (10 mM Tris–HCl, pH 7.4, 150 mM NaCl, 2% Triton X-114, 1× Protease Inhibitor Cocktail). Lysed parasites were centrifuged at 8,800*g* for 10 min at 0 °C to remove cell debris. The pellet (P) was solubilized in Lämmli buffer. The supernatant was incubated at 37 °C for 10 min to induce phase separation and centrifuged at 3,000*g* for 3 min. The upper phase (aqueous phase, AP1) was removed and stored on ice. The lower phase (detergent-rich phase, DP) was mixed with 1 ml of buffer A (10 mM Tris–HCl, pH 7.4, 150 mM NaCl, 0.06% Triton X-114, 1x Protease Inhibitor Cocktail) incubated at 10 °C for 10 min and subjected to a new phase separation at 37 °C (Supplementary Fig. 1A). The aqueous phase (AP2) was removed and the extraction was repeated once more with 1 ml of buffer A. The three aqueous phases (AP1–3) and the final detergent-rich phase were precipitated by adding 3 vol. of cold acetone at −20 °C for 30 min and then centrifuged at 12,000*g* for 20 min. Pellets were dried on the bench and solubilized in 20 μl of Lämmli buffer at 70 °C for 5 min. Samples were run approximately 2 cm into a 12% standard size SDS–polyacrylamide gel (termed short gel). The experiment was repeated with small modifications. Proteins from the detergent-rich phase were solubilized in Lämmli buffer at 37 °C for 15 min with short cycles of vortexing every 5 min. The gel was run at approximately 4 cm to obtain a better protein separation (termed long gel). In Supplementary Table S1 which lists all identified proteins, AP1 refers to the aqueous phase after the first phase separation and DP1 and DP2 refer to the final detergent phase obtained in the first and second experiment, respectively.

### 2.4 Identification and bioinformatic analysis of proteins

#### 2.4.1 LC-MS/MS analysis

Frozen samples were thawed at RT and centrifuged at 20,000*g* for 25 min. Thirteen microlitres of supernatant was transferred to a 96 well plate and mixed with 3 μl of 2.6 M formic acid to reduce evaporation.

The peptide mixtures were then analysed on a LC-MS/MS platform which contained an LTQ ion-trap mass spectrometer (Thermo-Electron, Hemel Hempstead, UK) coupled on-line to a Dionex Ultimate 3000 (Dionex Company, Amsterdam, The Netherlands) HPLC system as previously described (Xia et al., 2008). The data associated with this manuscript may be downloaded from ProteomeCommons.org (https://proteomecommons.org/data-search.jsp, Tranche hash:

3idGhZ+gXOGy4LXCSnvjqQShoEJCAy9HcfBWsYFKJmCma05n+6t97jj130XsJybg0Xwn4GSj3GUdS+2wWQkJucvojF4AAAAAAAA7Ew==.

#### 2.4.2 Raw data transformation

The resulting MS/MS spectra were submitted to Mascot (Matrix Science, (Perkins et al., 1999)) and searched against *T. annulata* (*Theileria*_prots) and *Bos taurus* (Bovine_IPI) annotated proteins. Fixed Mascot search parameters were: carbamidomethyl modification of cysteine (C); variable oxidation of methionine (M); peptide tolerance ±1.5 Da; MS/MS tolerance ±0.8 Da; +1, +2, +3 peptide charge-state; single missed trypsin cleavage, decoy: yes; report top: auto hits. Proteins with a score below 50 were removed from the list in order to avoid false positive hits. The identified proteins were compared with macroschizont, merozoite and piroplasm EST information on GeneDB (Pain et al., 2005). Lists of all macroschizont, merozoite and piroplasm-expressed genes were obtained with the keyword search for a specific stage.

#### 2.4.3 Transmembrane domain (TMD), signal peptide/anchor and GPI-anchor signal prediction

TMD, signal peptide/anchor and glycophosphatidylinositol (GPI)-anchor predictions are annotated for all proteins on GeneDB. TMDs were predicted by TMHMM2.0 (Krogh et al., 2001), signal peptide/ anchor by SignalP 2.0 HMM (Nielsen et al., 1997a,b) and GPI-anchor signal by DGPI v2.04. Lists of all proteins with a prediction were downloaded with the Complex/Boolean Query function on GeneDB.

All identified proteins were assigned after the MIPS FunCat (Functional Catalogue) (Ruepp et al., 2004). If possible, proteins were categorised according to the biological process annotation on GeneDB. If no biological process was annotated on GeneDB, but a molecular function was available, the corresponding Gene Ontology (GO) number of the function was sent to MIPS FunCat. If neither a biological process nor a molecular function was available on GeneDB, proteins were classified with the help of annotated motives/domains (Pfam, InterPro), protein names or homologues. *Theileria annulata* protein sequences were sent to NCBI BLAST to identify homologous proteins. GO annotations from homologues were only trusted if the *E*-value was lower than 1e−25.

#### 2.4.4 Classification of subcellular localisation

Very few proteins have a predicted subcellular localisation annotated on GeneDB. Therefore all proteins were classified with the help of bioinformatic tools according to a putative localisation. If possible, proteins were classified according to their FunCat annotation since some processes/categories are localisation-specific. Unclassified proteins were then categorised with help from the protein name, homologues and motives/domains. For the rest of the proteins, prediction programs such as WoLF PSORT (Horton and Nakai, 1999), TargetP (Emanuelsson et al., 2000), Plasmit (Bender et al., 2003), PlasmoAP (Foth et al., 2003) and Pats (Zuegge et al., 2001) were used. To avoid incorrect annotations, all proteins without a clear result were added to the category: not classified/multiple localisation.

## 3 Results and discussion

### 3.1 Analysis of whole parasite lysate

The analysis of WPL by 1-D gel LC-MS/MS resulted in 645 non-redundant proteins, with a score of 50 or higher, representing 17% of the 3,792 proteins predicted in the proteome of all life cycle stages of *T. annulata*. A list of all proteins identified in this study is provided in Supplementary Table S1 and can also be accessed via EuPathDB (www.eupathdb.org). These results are in line with observations made for *Cryptosporidium parvum* for which 16% of ∼3,900 predicted proteins were identified in the sporozoite life cycle stage (Sanderson et al., 2008). In a similar study on *Toxoplasma*, 939 of ∼7,800 (∼12%) predicted proteins were identified with 1-DE gel LC-MS/MS (Xia et al., 2008).

For most proteins identified in proteomic studies on *Toxoplasma gondii*, *Plasmodium falciparum* or *Neospora caninum*, corresponding ESTs were found with high frequency (88.2%, 84.2% and 72.6%, respectively). For 372 of the proteins identified (58%) in the present analysis, no corresponding ESTs were found in the macroschizont EST library (GeneDB). For 100 proteins (16%), the corresponding EST was present in libraries prepared from merozoites and/or piroplasms, but not from macroschizonts. For 173 proteins (27%), the corresponding ESTs were lacking altogether. Such discrepancies have also been observed in other apicomplexan parasites where examples exist of readily detected proteins whose corresponding genes display little or no detectable transcription (Wastling et al., 2009). With high-throughput sequencing data now becoming more readily available the situation is much improved; for example, recent mRNA deep sequencing for *T. gondii* and *N. caninum* (Reid et al., 2012) indicates that transcripts can be detected for nearly all proteins identified by MS in these organisms. It is important to note, however, that this does not mean that the correlation between transcript abundance and protein abundance is linear; on the contrary this complex relationship often leads to notably apparent discrepancies reflecting a range of factors such as the rate of protein and/or mRNA turnover (Wastling et al., 2012).

Proteins reported to be expressed by *T. annulata* merozoites or piroplasms could also be detected. The cultures we use consist of continuously proliferating bovine macrophages, which harbour the transforming schizont and have been maintained for many years in the laboratory. Merogony is a stochastic process known to occur at low levels in cultures of *T. annulata*-transformed cells. In established cultures the process is inefficient but merogony can be induced further by increased temperature or different types of stress, eventually resulting in the production of mature merozoites (Shiels et al., 1992; Schmuckli-Maurer et al., 2008).

The presence of piroplasms, which develop solely in red blood cells upon merozoite invasion, can be excluded under the culture conditions that were used. There was also no obvious morphological evidence for the presence of merozoites. The most likely explanation for the presence of proteins encoded by genes reported to be expressed by *T. annulata* merozoites or piroplasms is therefore that some parasites stochastically undergo early merogony.

Interestingly, proteins previously reported to be expressed by *Theileria* sporozoites were also identified. As the schizont-infected cells have been maintained for several years in culture, the presence of sporozoites can be excluded. TA17375 (GeneDB) is a *T. annulata* ortholog of the *T. parva* ‘polymorphic antigen precursor’ also called p150 (UniProt Accession Number Q27028) (Skilton et al., 1998), a sporozoite protein that contains a proline-rich region and was shown to cross-react with a *T. parva* polymorphic immunodominant molecule (PIM). In this context, p104, a microneme-rhoptry protein expressed in sporozoites, was also identified in Triton X-114 extracts (see Section 3.2). In this case, we posit that these proteins continue to be expressed by the parasite after invasion and differentiation to the schizont stage.

In our analysis of WPL, only 10.9% of the identified proteins contained a predicted TMD, which contrasts with the 22.2% predicted for all proteins. In macroschizonts, 20.9% of the genes for which transcripts could be detected were predicted to contain TMD, indicating that there is no bias towards low expression of such genes for this life cycle stage. A similar observation was made for proteins containing a signal peptide/anchor. Proteins containing a signal peptide are interesting candidates for host pathogen interactions as they may be secreted into the host cell, and parasite proteins anchored in the plasma membrane have the potential to interact with host cell components. Whereas 14.7% of all predicted proteins and 13.1% of the proteins with corresponding macroschizont ESTs are predicted to contain a signal peptide/anchor, this only applies to 7.9% of the proteins found in our analysis. Only nine, four and three proteins were detected containing two, three or four TMDs, respectively. The fact that proteins with multiple TMDs appeared to be strongly under-represented in the list of identified proteins detected in WPLs pointed towards a technical explanation. Indeed, in previous proteomics studies on *Toxoplasma* and *Cryptosporidium*, the ratio of identified and predicted TM proteins was similar to that observed in this study and most of the proteins with multi-TM helices were first identified when a multidimensional protein identification technology (MudPIT) analysis was performed.

### 3.2 Analysis of Triton X-114 lysates

**Fig. 1:**
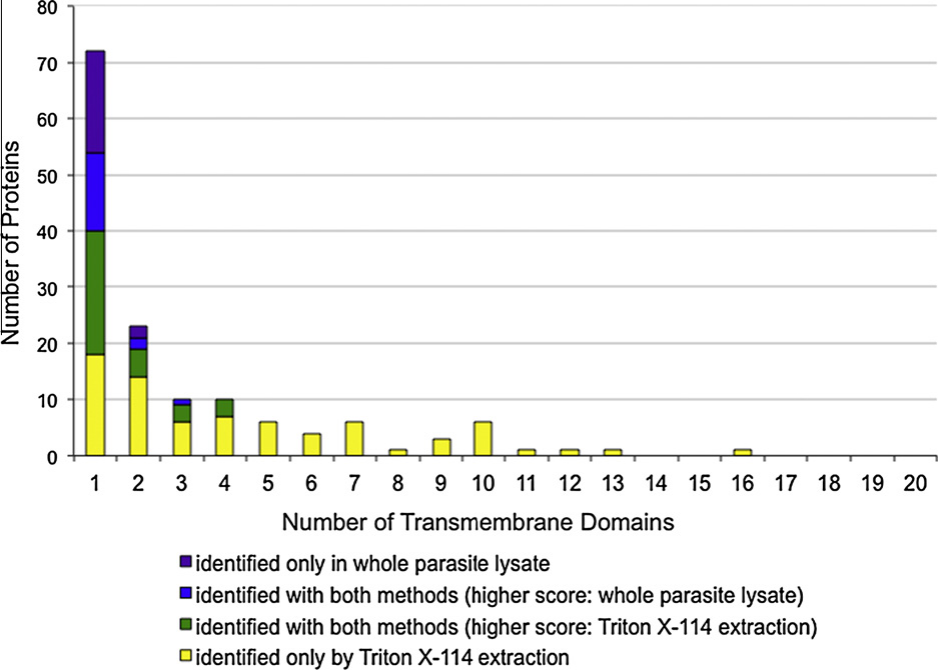
Triton X-114 extraction enhances the identification of proteins with transmembrane (TM) domains. Seventy-five additional *Theileria annulata* TM proteins were identified and proteins with more than five TM domains were only observed in the Triton X-114 detergent-rich phase.

Considering the paucity of TM proteins, it was decided to bias the protein extraction procedure in favour of membrane proteins and extracted purified schizonts using the detergent Triton X-114 (Bordier, 1981) as described by Cordero et al. (2009), with some minor modifications (Supplementary Fig. S1A). As expected, in 1-D gel analysis the protein pattern of the aqueous and detergent phases differed significantly (not shown). Proteins contained in the detergent-rich phase were subjected to 1-D gel SDS–PAGE and the corresponding lane cut into 30 continuous slices, digested with trypsin and analysed by LC-MS/MS. Using this extraction procedure, 762 proteins with a Mascot score >50 were identified, 459 of which were non-redundant. Among these, 153 had not been detected in WPL and 75 of these were predicted to have at least one TMD. In total 125 TM proteins were found in the detergent enriched fraction, almost twice the number found in the analysis of WPL (Fig. 1) and the relative number of TM proteins was increased from 10.9% (70 of 645 proteins) to 27.2% (125 of 459). Two-thirds of the proteins found in both samples had a higher score in the Triton X-114 extracts. Using Triton X-114 extraction, TA08425 (GeneDB), a *T. annulata* ortholog of the *T. parva* microneme-rhoptry antigen (also called p104; UniProt Accession Number Q962G6) was also identified. This finding is particularly interesting, as p104 was recently found to participate in parasite/host cell microtubule interactions (unpublished data from our laboratory).

Fractionation by Triton X-114 also helped to identify proteins with multiple TMDs (5–16), including different transporters. Interestingly, with a share of 59.5%, proportionately more unclassified proteins were newly identified upon detergent extraction than was the case for WPL (34.9 %). Taken together, these findings argue in favour of analysing Triton X-114 extracts in addition to WPL to expand the repertoire of identified proteins, in particular membrane-bound proteins.

### 3.3 Classification of identified proteins

Using the different lysis and extraction methods, a total of 812 proteins were identified, representing 21.4% of the predicted *T. annulata* proteome (see Supplementary Table S1). Different heat shock proteins, actin, glyceraldehyde-3-phosphate dehydrogenase, enolase and elongation factor belonged to the most readily detected proteins. This is in agreement with observations made in a preliminary analysis of the *T. parva* schizont proteome using a combination of high-resolution 2-D gel electrophoresis and MS (Bishop et al., 2009).

**Fig. 2:**
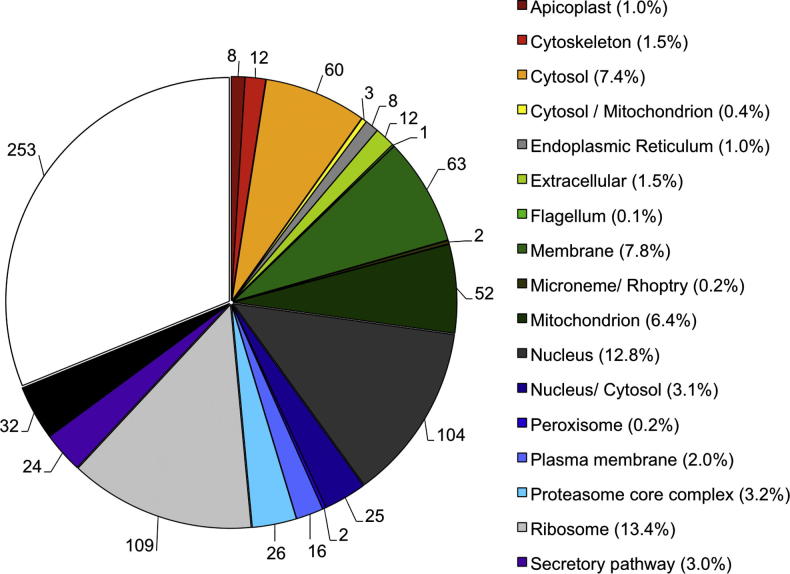
Subcellular localisation of all identified *Theileria annulata* proteins (whole parasite lysate (WPL) + Triton X-114 extraction). Actual numbers for each category are presented on the pie graph.

A set of algorithms was used to classify proteins based on predicted subcellular localisation (Fig. 2). Whereas proteins allocated to the cytoplasm, mitochondrion, nucleus and ribosome were prominent, for 29.8% of the proteins a subcellular localisation could not be allocated.

**Fig. 3:**
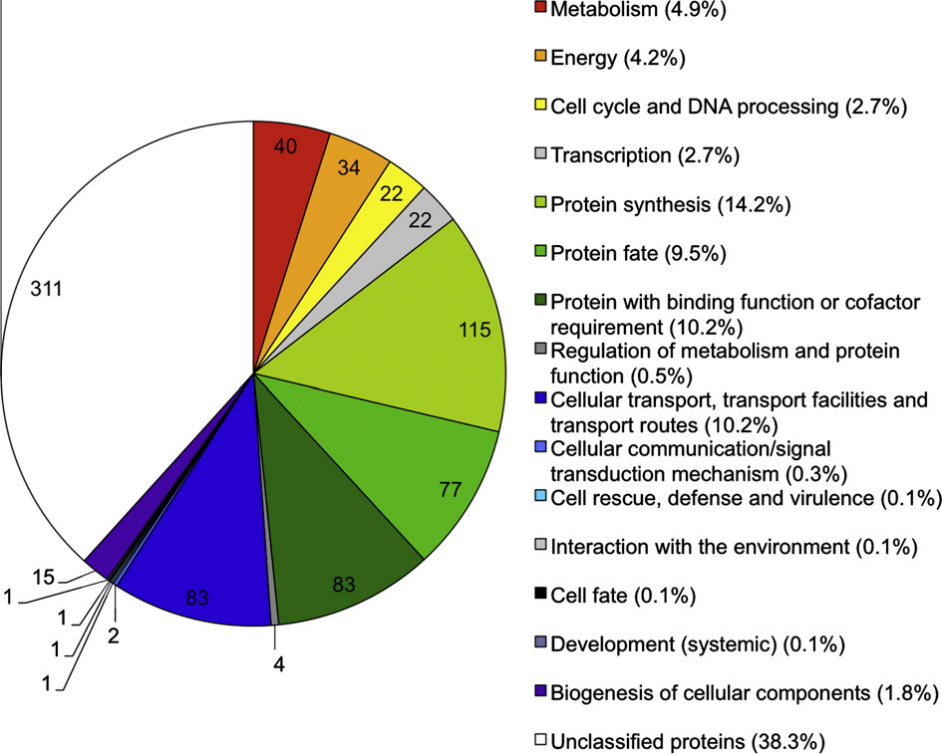
MIPS Functional Catalogue (FunCat) assignment of all identified *Theileria annulata* proteins (whole parasite lysate (WPL) + Triton X-114 extraction). Actual numbers for each category are presented on the pie graph.

All identified proteins were assigned to a MIPS FunCat Category (Fig. 3). With 38.3%, the unclassified proteins presented the largest category. A large category contains proteins involved in protein translation (14.2%). Other large categories involved proteins that regulate protein folding, modification, processing (together 19.7%) and cellular transport (together 10.2%). Proteins with functions assigned to cellular transport were under-represented in WPL analysis, but increased from 44 to 83 when data obtained from WPL and Triton X-114 extraction were pooled.

### 3.4 Glycolysis and citric acid cycle

**Fig. 4:**
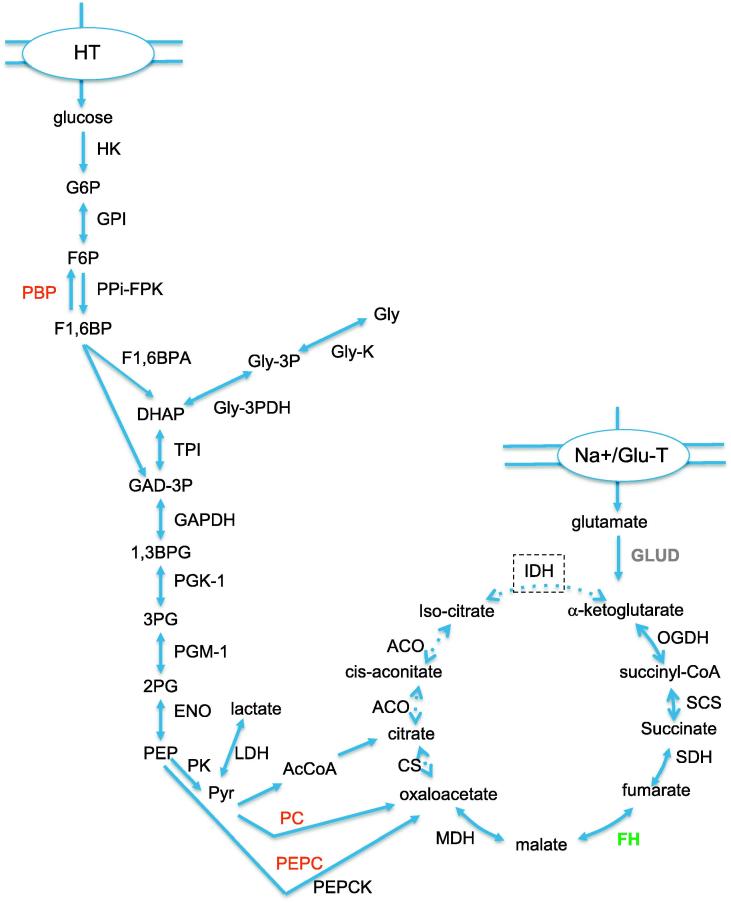
Almost all *Theileria annulata* proteins involved in glycolysis and citric acid cycle were identified in the study. The glycolysis pathway is shown on the left and the citric acid cycle is shown on the right. Reaction products: G6P, glucose 6-phosphate; F6P, fructose 6-phosphate;F1, 6BP, fructose 1,6-bisphosphate; DHAP, dihydroxyacetone phosphate; Gly-3P, glycerol 3 phosphate; Gly, glycerol; GAD-3P, glyceraldehyde 3-phosphate; 1, 3BPG, 1,3-bisphosphoglycerate; 3PG, 3-phosphoglycerate; 2PG, 2-phosphoglycerate; PEP, phosphoenolpyruvate; Pyr, pyruvate; AcCoA, acetyl coenzyme A. Enzymes with identified *Theileria annulata* proteins included in brackets: HK, hexokinase (TA19800); GPI, glucose 6-phosphate-isomerase (TA04045); PPi-FPK, pyrophosphate dependent phosphofructokinase (TA13950); PBP, fructose bisphosphatase; F1, 6BPA, fructose bisphosphate aldolase (TA20060); TPI, triosephosphate isomerase (TA08590); Gly-3PDH, glycerol-3-phosphate dehydrogenase; Gly-K, glycerol kinase; GAPDH, glyceraldehyde phosphate dehydrogenase (TA08145, TA15530); PGK, phosphoglycerate kinase (TA06655); PGM, phosphoglycerate mutase (TA10465); ENO, enolase (TA10425); PK, pyruvate kinase (TA11540, TA10915); LDH, lactate dehydrogenase (TA09590); PC, pyruvate carboxylase; ACO, aconitase (TA17020); IDH, isocitrate dehyrogenase (TA10850); OGDH, oxoglutarate dehydrogenase (TA05275, TA08530); SCS, succinyl coenzyme A synthetase (TA02815, TA10625); SDH, succinate dehydrogenase (TA19430, TA03455); FH, fumarate hydratase; MDH, malate dehydrogenase (TA18100); CS, citrate synthase (TA14450); PEPC, phosphoenolpyruvate carboxylase; PEPCK, phosphoenolpyruvate carboxykinase (TA20590); GLUD, glutamate dehydrogenase (TA11105) (Voet et al., 2006). Transmembrane transporters: HT, Hexose transporter (TA02480); Na^+^/Glu-T, sodium glutamate symporter (TA10315). Green indicates proteins that were not identified, but predicted, red shows no prediction and grey indicates proteins that were identified and are also likely to be involved. Dashed arrows in the citric acid cycle indicate that only a branch of the cycle is active in the schizont stage.

Knowledge on the central carbon metabolism of *Theileria* is very limited compared with other apicomplexan parasites such as *Plasmodium* (Olszewski and Llinas, 2011) and *Toxoplasma* (Polonais and Soldati-Favre, 2010). A comprehensive comparison of apicomplexan metabolic pathways is also available through http://www.llamp.net. The glycolysis and citric acid cycle rely on conserved proteins that are found in almost all eukaryotic cells (Voet et al., 2006). The genes for all 10 enzymes of glycolysis are present in the genomes of apicomplexans such as *Toxoplasma, Plasmodium*, *Theileria* and *Babesia.* In our analysis, all corresponding enzymes were detected (Fig. 4 and Supplementary Table S2). A transmembrane hexose transporter was also identified that could contribute to glucose import from the host cell, as shown for *Plasmodium* (Slavic et al., 2010). For these proteins, the average/median score, number of peptide matches and sequence coverage is significantly higher than for all other identified proteins, indirectly indicating that these proteins are abundant. Our observations are in line with the work of Kiama et al. (1999) who measured enzymatic activities in order to assess the significance of glycolysis in *T. parva* schizonts. In this work, it was also found that the enzymatic activities of glycerol kinase and glycerol 3-phosphate dehydrogenase were approximately 16 times lower than that of the other enzymes. Together, these results point towards a functional glycolytic pathway in *Theileria* schizonts, with low levels of glycerol catabolism. The fructose bisphosphatase gene, encoding an enzyme required for gluconeogenesis, cannot be found in the *Theileria* genomes. On the other hand, phosphoenolpyruvate carboxykinase (PEPCK), an enzyme that converts oxaloacetate to PEP, thus supplying citric acid cycle-derived carbon for gluconeogenesis, is expressed. PEP carboxylase, which essentially runs the reverse reaction, however, is absent.

Aerobic glycolysis is linked to the citric acid cycle via acetyl-CoA. Interestingly, not all of the genes encoding subunits of the pyruvate decarboxylase, which catalyses the reaction of pyruvate to acetyl-CoA and CO2, were found in the *Theileria* genome (Gardner et al., 2005; Pain et al., 2005). An alternative route to the citric acid cycle is the carboxylation of pyruvate to oxoglutarate, which is catalysed by pyruvate carboxylase, but this enzyme is also not present in *Theileria*. Despite this, a form of citric acid cycle was proposed to be present in *Theileria* (Gardner et al., 2005). All enzymes/subunits predicted from the genome were identified by MS, except for fumarate hydrase which was, however, represented in the EST library. In *T. parva* schizonts, the activities of most of the enzymes of the citric acid cycle were found to be very low, and only a branch of the citric acid cycle involving malate dehydrogenase, fumarase and succinate dehydrogenase appears to be active, potentially operating in the reverse direction of the citric acid cycle to synthesise succinate (Kiama et al., 1999). In our analysis, the number of peptide matches of the citric acid cycle enzymes was much lower compared with glycolytic enzymes. It was suggested that glutamate could be a supplementing intermediate for the citric acid cycle (Gardner et al., 2005). Glutamate can be converted into α-ketoglutarate by glutamate dehydrogenase. The latter was identified with a very high score. Glutamate might thus be a crucial intermediate and this hypothesis is underpinned by the identification of a sodium-glutamate symporter.

Incorporation studies on *Babesia rodhanini* also demonstrated the absence of a complete citric acid cycle (Rickard, 1970). In the case of *Plasmodium*, it has been proposed that, despite the presence of all enzymes, at least the asexual stages do not rely on the citric acid cycle for energy generation (van Dooren et al., 2006). However, recent studies involving transcriptomic, proteomic and metabolomic analyses suggest this model may be too simple (reviewed in Polonais and Soldati-Favre, 2010); the citric acid cycle may be working bidirectionally and be more prominent in different life cycle stages. This also applies to *Toxoplasma*, in which genes for all of the enzymes of the citric acid cycle are present and enzymes appear to be targeted to the mitochondrion (Fleige et al., 2008; Xia et al., 2008).

Taken together, it would appear that the anaerobic pathway is the main route of glucose metabolism in *Theileria* macroschizonts, that gluconeogenesis does not occur, glycerol catabolism occurs at low levels and that only a branch of the citric acid cycle is active (Kiama et al., 1999) in a process that does not participate in energy generation in the schizont. Additional studies will be required to determine whether other stages in the *Theileria* life cycle yield energy from oxidative phosphorylation .

### 3.5 Proteins involved in host/parasite interactions

The schizont is not contained in a parasitophorous vacuole and resides free in the cytoplasm where it interacts with host cell microtubules (Seitzer et al., 2010; von Schubert et al., 2010). A number of proteins have also been proposed to be secreted into the host cell cytoplasm from where they could interfere with host cell signalling pathways or translocate into the nucleus where they could modulate host cell gene expression, potentially contributing to transformation.

TaSP (the *T. annulata* ortholog of *T. parva* PIM), is abundantly expressed on the schizont surface and has been proposed to interact with host cell microtubules (Seitzer et al., 2010); this also applies to TaSE (GeneDB, TA20205). Surprisingly, neither of these proteins could be detected by MS.

Further, members of the family of subtelomere-encoded variable secreted proteins (SVSPs) (Gardner et al., 2005; Pain et al., 2005) and TashATs, which contain AT-hook DNA-binding domains and have been reported to localise to the nucleus of *T. annulata*-transformed cells (Swan et al., 2001), were not detected. With 85 members in *T. parva* and 48 members in *T. annulata*, SVSP genes constitute the largest family observed in these organisms. The general structure of SVSPs consists of a short conserved N-terminal region, in most cases containing a putative signal peptide for secretion, followed by a QP-rich region, which is predicted to be highly unstructured and a conserved C-terminus that has no significant identity to known proteins. In many SVSPs a nuclear localisation signal was also found. In *T. parva*-transformed cells, a large contingent of the SVSP gene family was found to be expressed at the RNA level (Bishop et al., 2005; Schmuckli-Maurer et al., 2009) and for at least one of the members, protein was also demonstrated using immuno-labelling techniques (Schmuckli-Maurer et al., 2009). TashATs form another intriguing family of parasite-secreted proteins, reported to translocate to the host cell nucleus. Whether these proteins contribute to transformation-specific changes in host cell gene expression is presently unknown. There could be several reasons why none of the SVSPs or TashATs was detected, including low abundance because proteins are systematically released from the schizont or unusual composition preventing detection. It is worth noting, however, that WPL samples for analysis were boiled up to five times in Lämmli buffer before they were subjected to SDS–PAGE. In this context, we recently observed that boiling schizont proteins often results in the formation of precipitates that remain in the stacking gel during SDS–PAGE. It cannot be excluded that this led to reduced detectability of certain proteins.

### 3.6 Concluding remarks

To our knowledge, we present the first characterisation of the *T. annulata* schizont proteome. This was greatly facilitated by the availability of a method to purify the parasite from the host cell cytoplasm and the application of Triton X-114 extraction for membrane protein isolation. This led to the identification of 812 proteins, most of which had only been predicted to exist based on genome and transcriptome analyses. The identification of proteins that were predicted to be expressed predominantly by the sporozoite, such as p150 and p104, is intriguing. It could point towards functions related to invasion, early establishment of the sporozoite upon entry, as well as long-term persistence of the schizont in the transformed cell. The failure to detect well-characterised proteins such as TaSP, or potentially secreted proteins such as SVSPs or TashATs, is surprising and potentially emphasises the technical limitations of the approach used in this study. With deep sequencing becoming widely accessible and increasingly affordable, and taking into account the continuous developments in quantitative proteomics, both the *Theileria* protein and transcript datasets are bound to expand and provide valuable tools for a system-wide approach to elucidate this unique host/parasite interaction.

## Appendix A Supplementary data

Supplementary data 2

Supplementary data 3 Overview of the Triton X-114 extraction. Purified *Theileria annulata* schizonts were subjected to Triton X-114 extraction as outlined in (A) and proteins separated by SDS–PAGE and stained with colloidal Coomassie (B). Lane P, all proteins contained in the pellet obtained after centrifugation of the cell lysate; AP1, AP2 and AP3, proteins present in the aqueous phase obtained after each phase separation; DP, all proteins in the detergent-rich phase. M: molecular weight markers in kDa.
