# Supplementary material for: Proteomic analysis of the Theileria annulata schizont
Source: Int J Parasitol. 2013 Feb;43(2):173–80. doi: 10.1016/j.ijpara.2012.10.017 (PMC3572392; doi:10.1016/j.ijpara.2012.10.017)
Supplement: Supplementary data 3 — Overview of the Triton X-114 extraction. Purified Theileria annulata schizonts were subjected to Triton X-114 extraction as outlined in (A) and proteins separated by SDS–PAGE and stained with colloidal Coomassie (B). Lane P, all proteins contained in the pellet obtained after centrifugation of the cell lysate; AP1, AP2 and AP3, proteins present in the aqueous phase obtained after each phase separation; DP, all proteins in the detergent-rich phase. M: molecular weight markers in kDa. [file mmc3.docx]

**Supplementary Fig. S1**

**A**

Purified *T. annulata*

Cell lysis and homogenisation

in 2% Triton X-114

Centrifuge to remove cell debris

Pellet (P1)

Transfer supernatant in new tube

incubation at -20°C for 24 h

phase separation 37°C

Aqueous phase (AP1)

Detergent-rich phase

Re-extraction with 0.06%

Triton X-114

phase separation 37°C

Detergent- rich phase (DP)

Aqueous phase (AP2, AP3)

Acetone precipitation

Acetone precipitation

Acetone precipitation

SDS-PAGE

Trypsin digest

LC- MS/MS

Mascot

repeat 1x

**B**

**
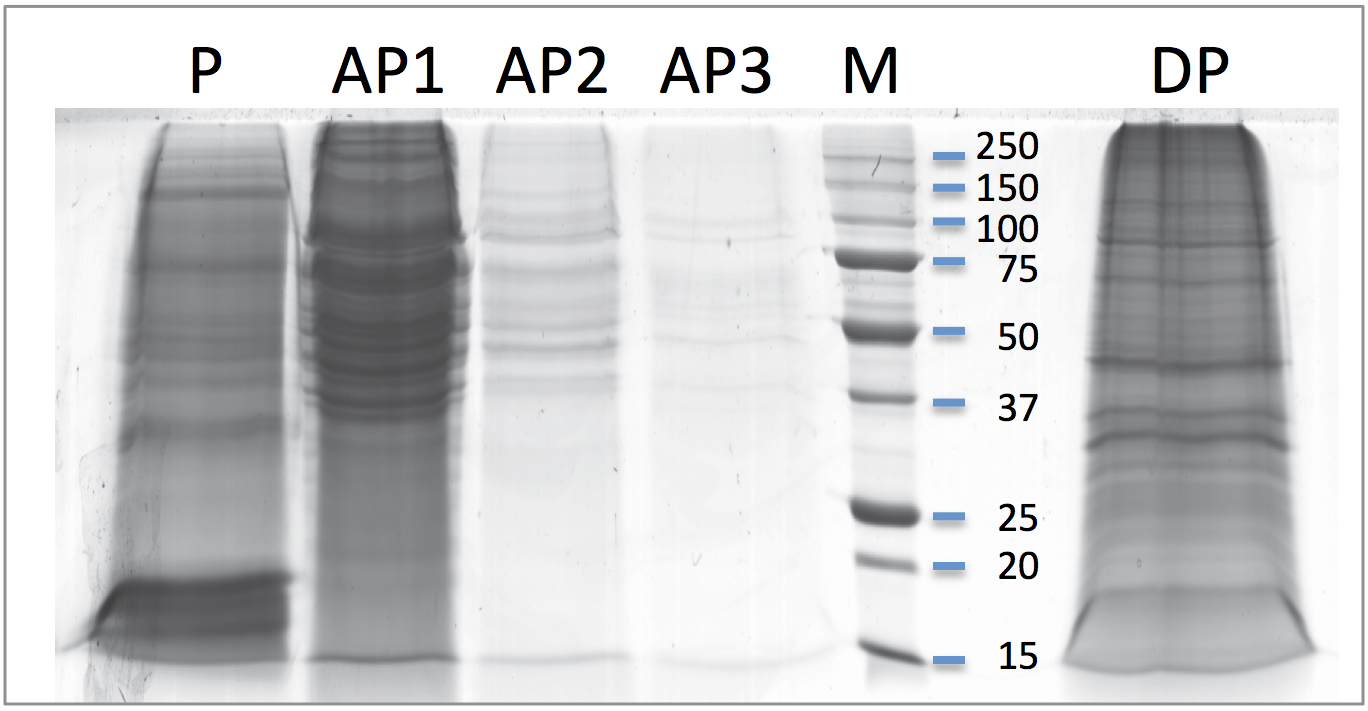
**
